# In-vitro assessment of coronary artery stents in 256-multislice computed tomography angiography

**DOI:** 10.1186/1756-0500-7-38

**Published:** 2014-01-14

**Authors:** Florian André, Dirk Müller, Grigorios Korosoglou, Waldemar Hosch, Hans-Ulrich Kauczor, Hugo A Katus, Henning Steen

**Affiliations:** 1 Department of Cardiology, University of Heidelberg, Im Neuenheimer Feld 410, Heidelberg 69120, Germany; 2 Philips GmbH Healthcare Division, Luebeckertordamm 5, Hamburg 20099, Germany; 3 Department of Diagnostic and Interventional Radiology, University of Heidelberg, Im Neuenheimer Feld 410, Heidelberg 69120, Germany

**Keywords:** CT angiography, Coronary artery stents, In-stent restenosis, Heart, Technology assessment

## Abstract

**Background:**

The important detection of in-stent restenosis in cardiovascular computed tomography (CT) is still challenging. The first study assessing the in-vitro stent lumen visualization of the state of the art 256-multislice CT (256-MSCT), which was performed by our research group, yielded promising results. As the applied technical approach is not suitable for daily routine, we assessed the capability of the 256-MSCT and its different reconstruction kernels for the coronary stent lumen visualization employing a clinically applicable technique in a phantom study.

**Results:**

The XCD kernel showed significantly lower artificial lumen narrowing (ALN) values (overall ALN < 40%) than the other reconstruction kernels (CC, CD, XCB) irrespective of the stent caliber. The ALN of coronary stents with a diameter >3 mm was significantly lower than of stents with a smaller caliber. The ALN difference between stents with a diameter of 3 mm and smaller ones was not statistically significant. Yet, the lumen visualization of the smaller stents was impaired by a halo effect. The XCD kernel showed more constant attenuation values throughout the different stent diameters than the other reconstruction kernels.

**Conclusions:**

The 256-MSCT provides a good lumen visualization of coronary stents with a diameter >3 mm. The assessment of stents with a diameter of 3 mm seems feasible but has to be validated in further studies. The clinical evaluation of smaller stents cannot be recommended so far. The XCD kernel showed the best lumen visualization and should therefore be applied in addition to the standard cardiac reconstruction kernels when assessing coronary artery stents using 256-MSCT.

## Background

In a previous study we could show that the 256-multislice computed tomography (MSCT) yields promising results for the visualization of coronary artery stents using a technical approach [1]. Therefore, we provide an update on this trial employing a clinically applicable technique.

Currently, percutaneous coronary intervention (PCI) and stent placement are the leading coronary revascularization therapies in patients with coronary artery disease (CAD). Approximately 492 000 patients underwent PCI procedures in the US in 2010 [2]. The clinical use of drug eluting stents (DES) instead of bare metal stents (BMS) reduces the rates of in-stent restenosis (ISR) from about 20-30% to less than 10% [3,4]. But despite the increasing amount of DES coronary interventions, ISR and stent thrombosis are still a major limitation of PCI, leading to an increased morbidity and mortality in patients with coronary stents [5]. Moreover, a considerable number of these patients have to undergo conventional coronary angiography (CA) to exclude ISR. In addition to bearing the risks of an invasive procedure, CA is linked to considerable health care costs. Hence, a non-invasive alternative may be of great clinical interest as well as of socio-economic benefit.

Nowadays, the angiography of the coronary arteries using MSCT is a non-invasive imaging modality for the reliable diagnosis and assessment of CAD yielding a sensitivity of 97% - 99% and a specificity of 88% - 89% [6,7]. Yet, the evaluation of coronary artery stents is challenging due to artifacts like beam hardening and partial volume effects caused by the metal of the stent struts and sporadically by the stents’ radio-opaque markers. First attempts to visualize the lumen of coronary artery stents using CT were made about 20 years ago using electron beam CT without reaching an image quality that was sufficient for analysis [8]. Since then, the MSCT technology has developed tremendously and new scanner generations with 4, 16 and 40 slices have been assessed for ISR detection [9,10]. With the introduction of the 64-MSCT the reliable visualization of ISR seemed to be achievable [11]. Yet, several reviews came to the conclusion that the diagnostic performance of the 64-MSCT was not sufficient in patients with implanted coronary artery stents [12-14].

The introduction of the latest CT scanner generations including dual source CT (DSCT) with high temporal resolution on the one hand and single-source MSCT with wide z-axis coverage of 256 or 320 slices on the other hand arose the hope for reliable stent lumen assessment. In addition, previous studies showed that stent-dedicated, sharper reconstruction kernels were able to reduce stent artifacts and improve stent lumen visualization [1,15,16].

In the first study using the 256-MSCT for the assessment of coronary artery stents, our research group got promising results [1]. We applied a full width at half maximum algorithm to measure the extent of the impaired in-stent lumen visualization objectively. Yet, this technical approach is associated with a high effort and is, therefore, impractical for a daily-base clinical use. Furthermore, the obtained results might reflect technical values rather than visual assessment as performed in clinical routine – hence the need for an assessment of the stent lumen visualization capability of the 256-MSCT using a clinically applicable approach. In this study, we sought to investigate coronary stent visualization and in-stent lumen assessment using various kernels in a state of the art 256-MSCT by employing a visual analysis technique.

## Methods

We included 51 stents of different diameters, materials, strut designs and manufacturers in this study. A summary is provided in Table 1.

**Table 1:** List of coronary artery stents

**Manufacturer**	**Name**	**∅ (mm)**	**L (mm)**	**Material**	**ST (mm)**	**Drug**	**ALN (%)**
Medtronic	Endeavor Resolute	2.25	12	CoCr	0.091	Zotarolimus	45
Medtronic	Endeavor Resolute	2.25	8	CoCr	0.091	Zotarolimus	44
Medtronic	Micro-Driver	2.25	24	Cobalt alloy	0.091		41
Abbott Vascular	Multi-Link Mini Vision	2.50	28	Cobalt alloy	0.081		41
Abbott Vascular	Multi-Link Mini Vision	2.50	18	Cobalt alloy	0.081		40
Biotronik	PRO-Kinetic	2.50	22	CoCr + SiC coating	0.060		53
Cordis	Cypher Select Plus	2.50	18	316 L	0.140	Sirolimus	43
Medtronic	Endeavor Resolute	2.50	12	CoCr	0.091	Zotarolimus	36
Medtronic	Endeavor Resolute	2.50	24	CoCr	0.091	Zotarolimus	32
Medtronic	Micro-Driver	2.50	24	Cobalt alloy	0.091		36
Terumo	Tsunami Gold	2.50	20	316 L	0.080		43
Abbott Vascular	Multi-Link Vision	2.75	8	CoCr	0.081		36
Medtronic	Endeavor Resolute	2.75	8	CoCr	0.091	Zotarolimus	33
Medtronic	Endeavor Resolute	2.75	30	CoCr	0.091	Zotarolimus	35
Medtronic	Endeavor Sprint	2.75	24	CoCr	0.091	Zotarolimus	33
Medtronic	Micro-Driver	2.75	18	Cobalt alloy	0.091		33
Medtronic	Micro-Driver	2.75	24	Cobalt alloy	0.091		36
B. Braun	Coroflex Blue	3.00	19	CoCr	0.065		41
B. Braun	Coroflex Please	3.00	19	316 L	0.120	Paclitaxel	40
Biotronik	PRO-Kinetic	3.00	30	CoCr + SiC coating	0.060		50
Boston Scientific	Liberté	3.00	20	316 L	0.097		38
Boston Scientific	Liberté	3.00	16	316 L	0.097		44
Boston Scientific	Taxus Liberté	3.00	16	316 L	0.097	Paclitaxel	39
Boston Scientific	Taxus Liberté	3.00	20	316 L	0.097	Paclitaxel	40
Cordis	Presillion	3.00	17	CoCr	0.073		38
Cordis	Presillion	3.00	12	CoCr	0.073		39
Medtronic	Driver	3.00	12	Cobalt alloy	0.091		40
Medtronic	Endeavor Resolute	3.00	12	CoCr	0.091	Zotarolimus	44
MSM	Experimental*	3.00	16	316 L + Tantal coating	0.080		38
Terumo	Tsunami Gold	3.00	18	316 L	0.080		42
Terumo	Tsunami Gold	3.00	18	316 L	0.080		39
Translumina	Yukon*	3.00	18	316 L	0.115		41
Translumina	Yukon Choice*	3.00	18	316 L	0.097		40
Translumina	Yukon Choice CC*	3.00	18	316 L	0.100		41
Abbott Vascular	Multi-Link Vision	3.50	28	CoCr	0.081		25
Abbott Vascular	Multi-Link Vision	3.50	12	CoCr	0.081		33
Biotronik	PRO-Kinetic	3.50	20	CoCr + SiC coating	0.080		27
Cordis	Cypher Select Plus	3.50	13	316 L	0.140	Sirolimus	32
Cordis	Cypher Select Plus	3.50	18	316 L	0.140	Sirolimus	30
Medtronic	Driver	3.50	9	Cobalt alloy	0.091		30
Medtronic	Endeavor	3.50	9	CoCr	0.091		30
Medtronic	Endeavor Resolute	3.50	24	CoCr	0.091	Zotarolimus	22
Medtronic	Endeavor Resolute	3.50	9	CoCr	0.091	Zotarolimus	25
Medtronic	Endeavor Sprint	3.50	24	CoCr	0.091	Zotarolimus	21
Medtronic	Endeavor Sprint	3.50	18	CoCr	0.091	Zotarolimus	25
Terumo	Tsunami Gold	3.50	10	316 L	0.080		33
Terumo	Tsunami Gold	3.50	18	316 L	0.080		30
Biotronik	PRO-Kinetic	4.00	20	CoCr + SiC coating	0.080		29
Medtronic	Driver	4.00	9	Cobalt alloy	0.091		29
Medtronic	Driver	4.00	24	Cobalt alloy	0.091		33
Medtronic	Driver	4.00	12	Cobalt alloy	0.091		31

*stent without a delivery system which was expanded using a balloon catheter.

List of different manufacturers, stent types, dimensions, materials and drugs of the 51 applied coronary artery stents. In addition, the ALN values of every stent using the XCD kernel are provided.

∅: nominal stent diameter.

L: stent length.

CoCr: cobalt chrome.

SiC: silicon carbide.

ST: strut thickness.

ALN: artificial lumen narrowing (given as percentage of the nominal lumen diameter).

The stents were assorted into three groups according to their diameters: group A: stents < 3 mm, group B: stents = 3 mm, group C: stents > 3 mm. The stents were expanded at the nominal pressure of their respective delivery system into a vessel phantom, which was made of plastic tubes with a wall thickness of 0.5 mm. Stents of group A and B were placed in tubes with an inner diameter of 3 mm, stents of group C in tubes with an inner diameter of 4 mm. The tubes were filled with contrast agent and saline (Ultravist 370, Bayer HealthCare, Leverkusen, Germany) providing a radio-density of approximately 250 HU. Afterwards, the tubes were closed at both ends and placed into a plastic basin. This was filled with an oil-iodine solution attaining a radiodensity of approximately -70 HU according to the epicardial fat tissue. The basin was placed into the gantry, thus aligning the tubes in a parallel manner to the scanner´s z-axis.

Imaging was performed on a 256-slice CT scanner (Brilliance iCT, Philips Healthcare, Cleveland, OH, USA) applying the following parameters: helical mode, collimation = 2 × 128 × 0.625 mm, tube voltage = 120 kV, tube current-time product = 800 mAs_eff_, pitch = 0.18, tube rotation time = 270 ms, field of view = 180 mm.

All images were reconstructed at 75% of an artificial ECG-signal with a heart rate of 60 bpm using a matrix of 512 × 512. Four different convolution kernels were applied: a) Xres detailed stent (XCD), b) cardiac sharp (CC), c) cardiac detailed stent (CD) and Xres standard (XCB). While XCB is the standard kernel for the reconstruction of coronary arteries, XCD and CD are dedicated kernels for the visualization of stents.

The analysis was carried out on a dedicated CT post-processing workstation (Extended Brilliance Workspace V 3.5.3.1020, Philips Healthcare, Cleveland, OH, USA) with a window center of 300 HU and a width of 1200 HU.

### Artificial lumen narrowing

Artificial lumen narrowing (ALN) was defined as the difference between the nominal stent diameter and the one measured visually using the electronic diameter provided by the workstation’s software. Measurements were performed in axial orientation in a proximal, middle and distal part of the stent and a mean value was calculated.

### Stent and tube lumen attenuation

As the measured radio-density inside the stent lumen can be influenced by artifacts like beam hardening or partial volume effects, we measured two values: stent lumen attenuation (SLA) and tube lumen attenuation (TLA). The quantification was carried out in a longitudinal view by a region-of-interest (ROI) technique. SLA was assessed by placing the largest possible ROI inside the lumen of the deployed stent omitting strut artifacts. For TLA two ROIs were positioned inside the tube lumen outside both sides of the stent and a weighted mean value was calculated.

### Attenuation and image noise

Attenuation was defined as the difference between stent lumen attenuation and tube lumen attenuation:

Attenuation=SLA-TLA

We defined image noise as the mean value of the standard deviations of three axial ROIs which were positioned outside the tube lumen in the oily fluid.

### Statistical methods

Data were analyzed with a statistical software (MedCalc Statistical Software, V 11.4.1.0, MedCalc bvba, Belgium). Normal distribution was assessed applying the Kolmogorov-Smirnov test. We used an analysis of variance for the comparison of the artificial lumen narrowing values of different groups.

As some of the attenuation values were not distributed normally, we applied the non-parametric Friedman test for the comparison of the different kernels. For the comparison of different stent size groups regarding the attenuation, the Mann–Whitney test was used as the stents of different groups were not paired.

Statistical significance was defined at p < 0.05 after application of the Bonferroni adjustment for multiple comparisons.

## Results

### Artificial lumen narrowing

The mean ALN values for the different reconstruction kernels and groups are listed in Table 2. In addition, a list of the ALN values of all stents as measured applying the XCD kernel is provided in Table 1.

**Table 2:** Artificial lumen narrowing

**Group**	**XCD**	**CC**	**CD**	**XCB**
All	36(21/53) ± 7	45(26/79) ± 11	45(29/71) ± 10	42(26/73) ± 10
A	39(32/53) ± 6	52(37/79) ± 12	51(39/71) ± 9	48(37/73) ± 10
B	41(38/58) ± 3	50(44/54) ± 3	49(36/57) ± 6	46(40/53) ± 4
C	29(21/33) ± 4	35(26/44) ± 5	34(29/46) ± 5	32(26/42) ± 5

Artificial lumen narrowing values are given as mean (minimum/maximum) ± standard deviation in percent of the nominal lumen diameter.

Collectively, the XCD kernel showed markedly lower ALN values than all other kernels (p < 0.001). Also the XCB kernel was significantly different to all other reconstruction algorithms (p < 0.001). Between the CC and CD kernel no significant differences were observed (p > 0.05).

ALN values for group A versus group B stents were not significantly different (p > 0.05) for all kernels in contrast to group C stents which showed a significantly lower value (p < 0.001).

**Figure 1:**
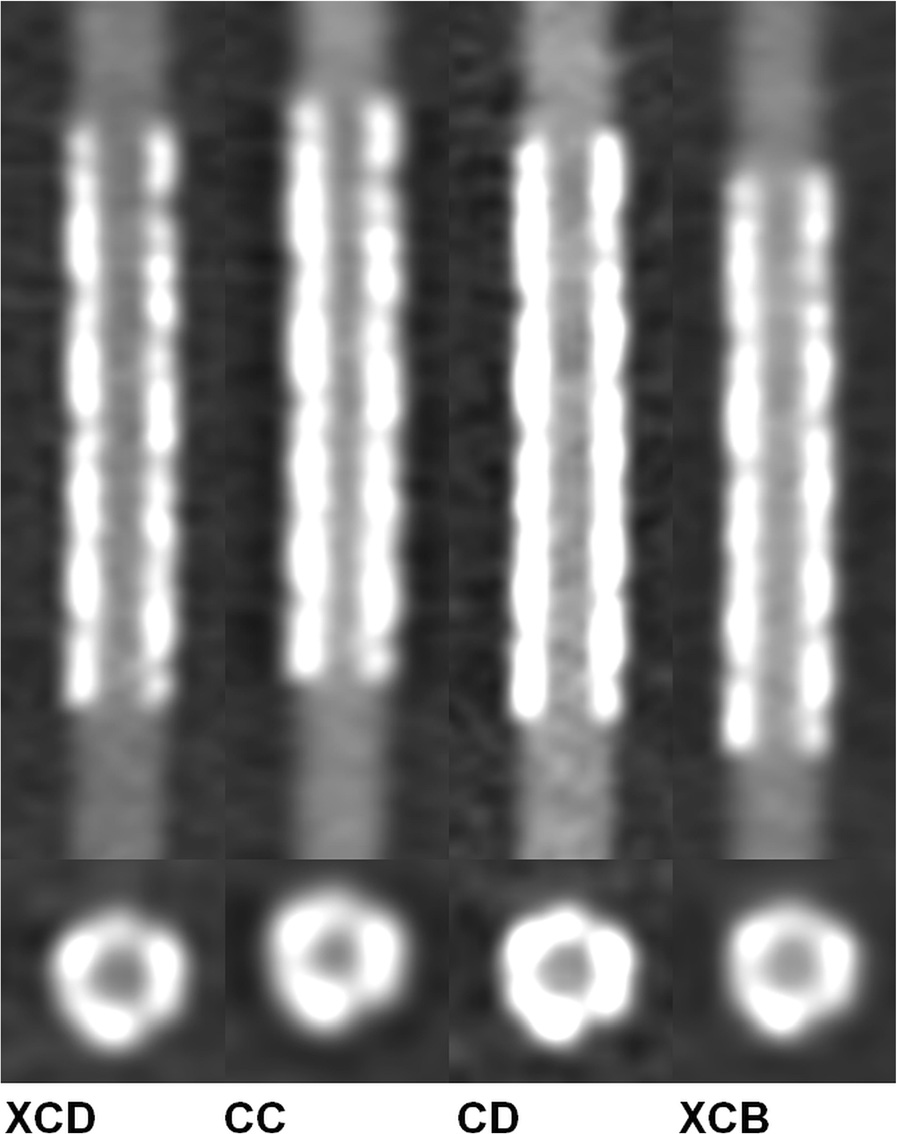
**Differences of reconstruction kernels.** Reconstructions of the same stent applying the four different kernels. (B. Braun Coroflex Please, nominal diameter 3.0 mm, length 19 mm).

**Figure 2:**
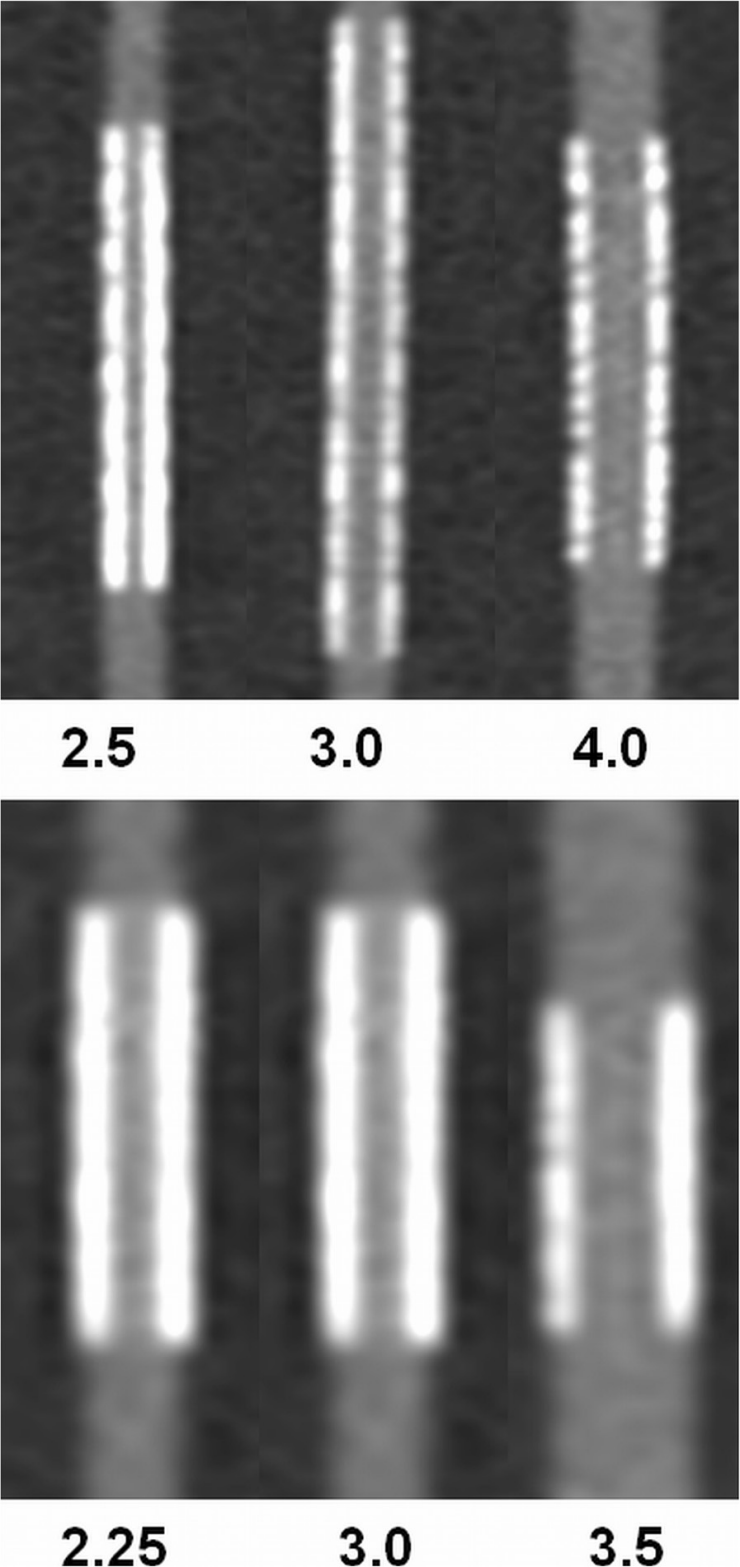
**Effect of different stent diameter.** Reconstructions of three different sizes of the same stent type (top: Biotronik PRO-Kinetic, bottom: Medtronic Endeavor Resolute). Diameter is given in mm. Zoom factors differ between the stent types.

Figure 1 shows an example of a stent reconstructed with various reconstruction kernels. Figure 2 demonstrates exemplarily the effect of the stent diameter by showing three different sizes of two stent types.

### Halo effect

**Figure 3:**
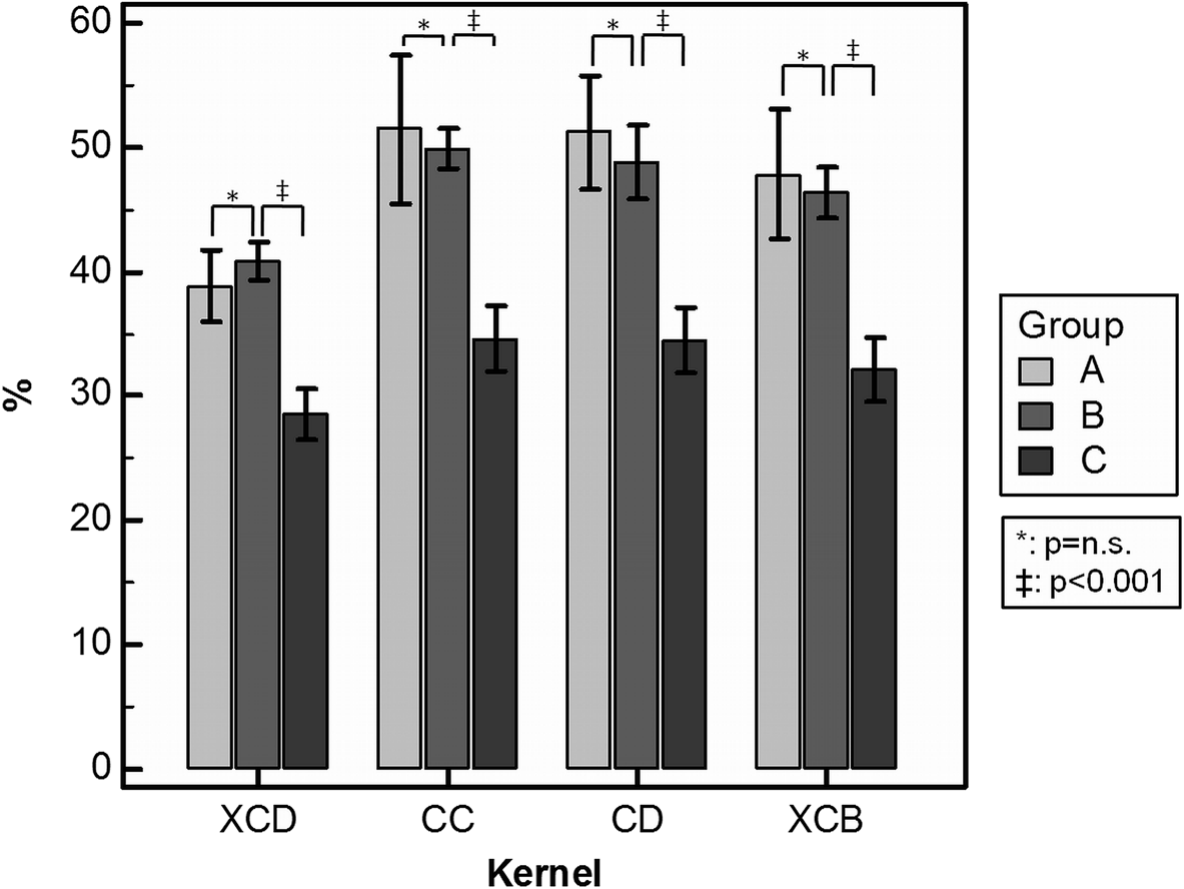
**Artificial lumen narrowing.** Comparison of the ALN values of different stent groups applying the four reconstruction kernels.

**Figure 4:**
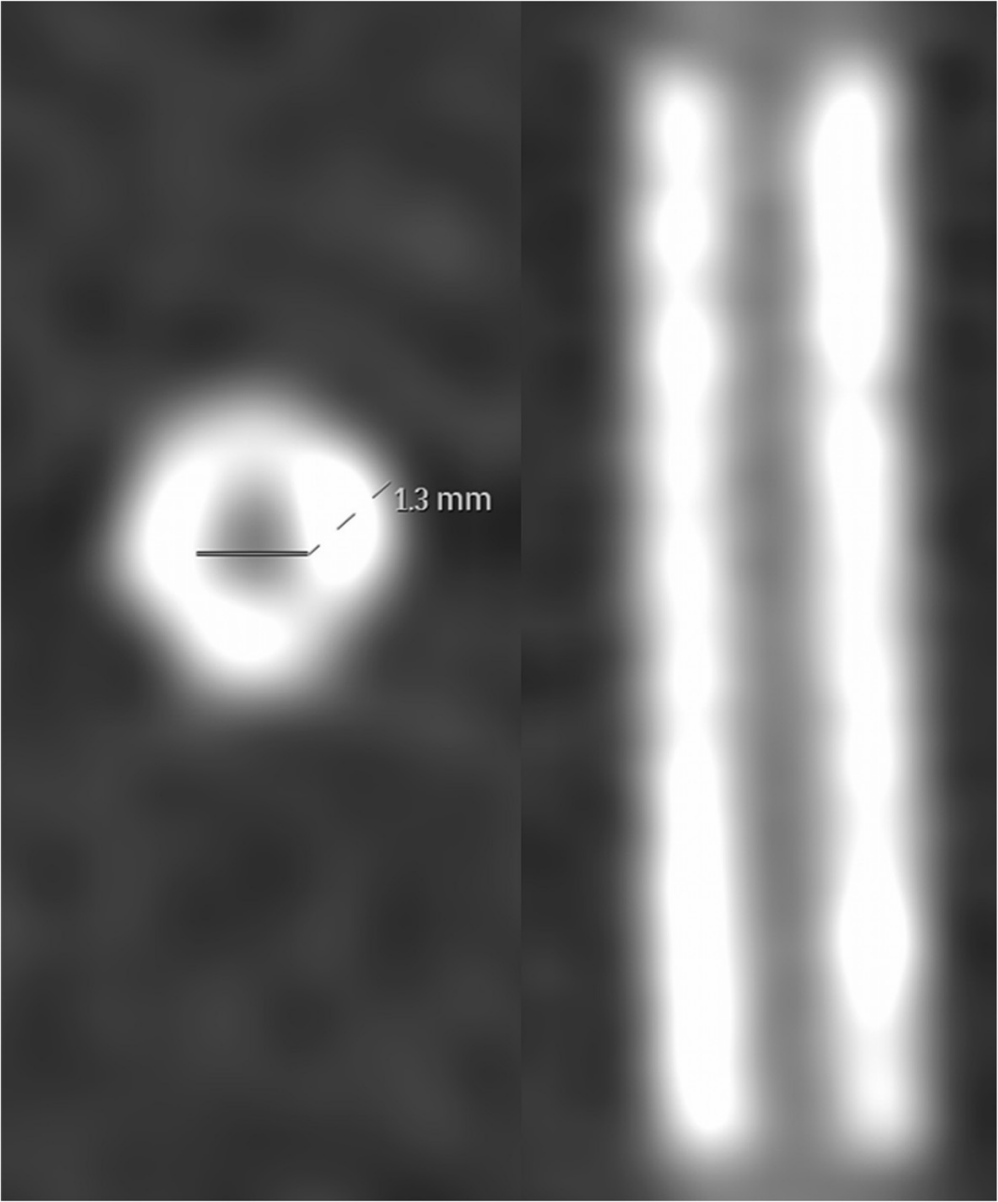
**Example of the halo effect.** The ALN for this stent was manually measured as 45%. However, the lumen visualization was considerably impaired by the halo effect (Medtronic Endeavor Resolute, nominal diameter 2.25 mm, length 12 mm).

Especially the XCD kernel yielded comparatively low ALN values for the small group A stents (see Figure 3). However, the apparently well-definable stent boundaries were visually hampered by what we labeled as 'halo effect’: an artificially increased in-stent lumen image signal in the vicinity of the strut-lumen interface that decreases gradually towards the stent lumen center. An example of the halo effect is given in Figure 4.

### Attenuation

The median attenuation values with the respective interquartile range (IQR) of all kernels and groups are summarized in Table 3.

**Table 3:** Attenuation

**Group**	**XCD**	**CC**	**CD**	**XCB**
All	43(22/174)_IQR_34	18(-177/392)_IQR_183	40(-78/254)_IQR_96	128(6/396)_IQR_122
A	55(22/174)_IQR_55	127(14/392)_IQR_186	78(14/254)_IQR_34	189(116/396)_IQR_158
B	41(27/125)_IQR_48	36(-2/151)_IQR_65	48(9/153)_IQR_56	131(87/265)_IQR_59
C	42(22/116)_IQR_17	-113(-177/-18)_IQR_35	-29(-78/33)_IQR_29	49(6/163)_IQR_33

Attenuation values are given as median (minimum/maximum) interquartile range in HU.

Collectively, the CC kernel (18 IQR 183 HU) showed significantly the lowest attenuation values followed by the CD kernel (40 IQR 96 HU). While the difference between CD and XCD (43 IQR 34 HU) yielded no significance, the XCB method showed a significantly higher value (128 IQR 122 HU) than all other kernels.

Consequently, we performed an analysis of the different stent size groups.

For group A stents, the discrepancy between all kernels was significant with the XCD kernel providing values closer to the optimum of zero than the other ones.

In group B, XCD showed no significant differences to CD whereas all other differences including those to XCB were significant. CC showed the best attenuation values followed by XCD.

In group C, only the difference between XCD and XCB was not significant with CD providing the attenuation values closest to the optimum of zero.

**Figure 5:**
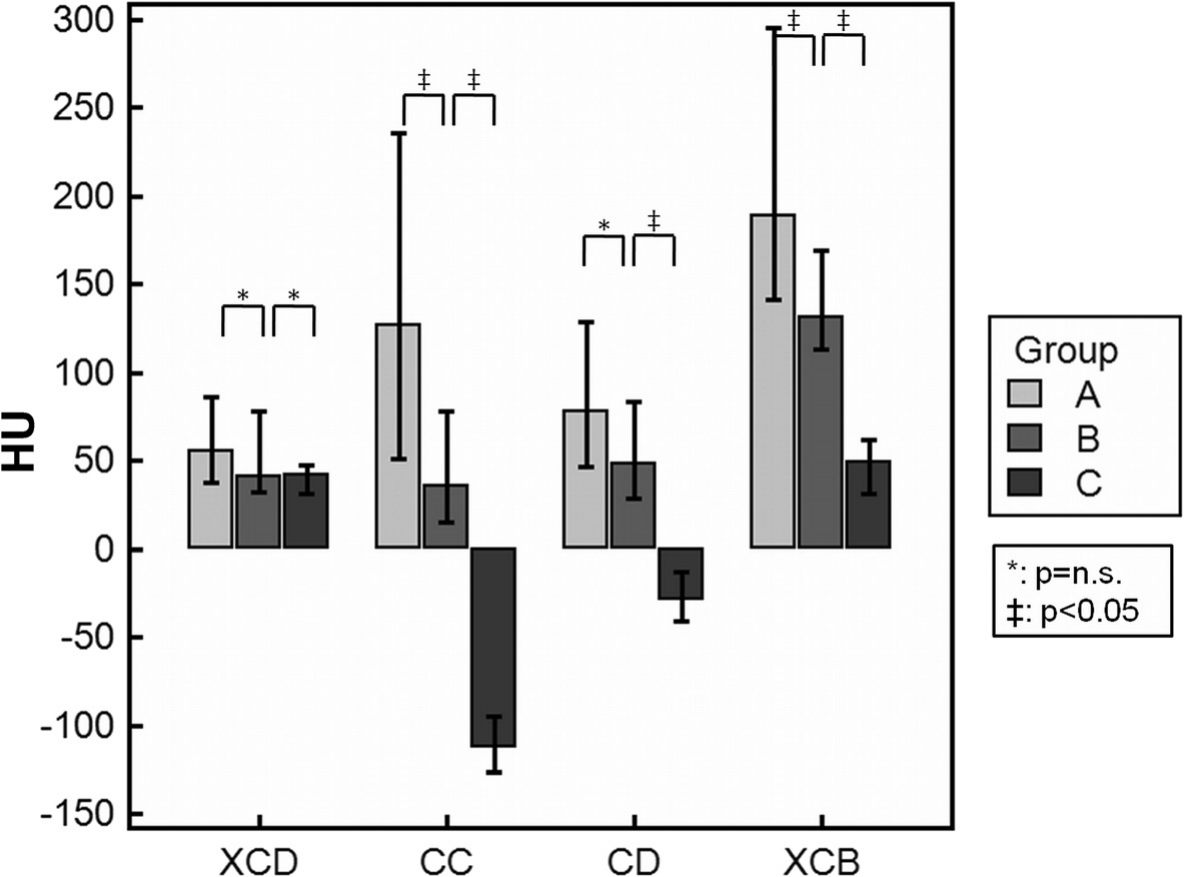
**Attenuation.** Attenuation values as provided by the different reconstruction kernels. Note that with increasing stent size the values of the CC and CD kernels become negative.

Whereas the values for XCD remained quite constant and only declined about 13 HU from group A to C, the other groups showed notably higher drops up to 240 HU for the CC kernel (see Figure 5). The CC and the CD kernel even showed negative values.

### Noise

As noise was measured independently from the stent diameter a sub-categorization into different groups was not reasonable. The soft XCB kernel showed significantly lower values than all other kernels (15.8 ± 1.9 HU). Of the remaining kernels, the CD kernel had the highest values (34.9 ± 4.6 HU), whereas values for the XCD (21.1 ± 2.9 HU) and CC (19.9 ± 2.2 HU) kernels were comparable. Yet the differences between all kernels were statistically significant (all p < 0.001).

## Discussion

In a state of the art 256-MSCT scanner four different reconstruction kernels were employed to scan 51 coronary artery stents. Recently published clinical studies could show that the quantification of atherosclerotic coronary lumen narrowing as well as the assessment of plaque is feasible with this scanner generation [17]. However, to date there is only scarce data regarding the potential of the 256-MSCT for the visualization of the coronary stent lumen [1,18,19]. In contrast to our first technical oriented studies, we applied a clinically applicable approach.

The applied XCB kernel is the standard algorithm for the assessment of coronary arteries in Philips CT scanners. For the reconstruction of coronary stents the CD and XCD kernel are provided by the manufacturer. Although both kernels have similar reconstruction characteristics, the XCD features a reduced image noise. In previous studies concerning stent visualization the B46f kernel has been recommended for the 64-MSCT and DSCT from Siemens [15,16] and the Q04 kernel for Toshiba’s 64-MSCT [20].

Overall and in all stent groups, the Philips XCD kernel showed lower ALN values than the other three kernels. The edge-enhancing characteristic of the XCD’s reconstruction method provided sufficiently visible strut margins, so that the electronic caliper could be positioned precisely in most measurements. Consequently, the ALN standard deviations of the XCD kernel were smaller compared to the other three kernels. A similar superiority of the XCD kernel became evident with respect to the attenuation. The measured attenuation values applying the XCD kernel were consistent across the stent size groups whereas the attenuation for the CC and CD kernels declined from highly positive values in small stents to even negative measurements in larger stents. The phenomenon of radiodensity values being lower in the stent than in the tube lumen has been described before [1,15,21]. One may speculate that the sharpening algorithms of these kernels could be responsible for this effect. However, the diagnostic value and interpretation of negative attenuation values within the stent lumen is unclear and needs further investigation. Although the CD kernel showed attenuation values closer to the optimum of zero in group C stents, it was accompanied by considerable higher noise and ALN.

As the XCB kernel is softer than the others, it provided significantly lower noise values. Yet, the noise difference between the XCD and the XCB kernels was only about 5 HU and therefore negligible.

In summary, the XCD kernel provided the most reliable ALN and attenuation values at the cost of a little higher noise. Our first conclusion therefore is that we recommend the application of the XCD kernel in addition to the routine XCB kernel when examining a patient with a coronary artery stent.

We investigated various coronary artery stents of different sizes, designs, materials and manufacturers. As most of the coronary stents used in clinical routine are either made of 316 L stainless steel or cobalt chrome, the stent size and architecture is, in a majority of cases, a more important factor for the quality of stent lumen visualization than the material.

At first glance, the ALN values for group A and B stents seemed to be astonishingly low with most values being <50% using the XCD kernel. However, the visibility of their lumina was impaired by a phenomenon we introduced as 'halo effect’ (Figure 4). This phenomenon resembles the so-called blooming artifact but unlike this it is rather characterized by altered attenuation values inside the stent than by an exaggerated thickness of the stent struts as described for blooming [22,23]. The halo effect seemed to be more pronounced in smaller stents, especially when reconstructed with harder kernels. Although the struts were clearly visible the assessability of the adjacent lumen was impaired. As the voxel signals at the interface between tube lumen and stent struts seemed to be artificially smeared, atherosclerotic processes like ISR could potentially be missed or misinterpreted. Clinically, the halo effect could therefore often lead to false diagnoses. Therefore, we advise against the employment of coronary CT for the exclusion of ISR in stents <3 mm due to the halo effect.

In agreement with our findings, in a lately published meta-analysis on 64-MSCT, Carrabba et al. stated that the rate of uninterpretable stent lumina tended to be more pronounced for stent diameters <3 mm [24]. Likewise, in a recent review about the diagnostic accuracy of 64-MSCT for the detection of ISR, Sun et al. concluded that stent lumen evaluation should be restricted to larger stents (>3 mm) [11]. Since these measurements were generated using 64-MSCT, state of the art scanner generations such as DSCT or 256-MSCT have encouraged the expectation that ISR in smaller stents could accurately be detected or excluded. And indeed, Pugliese et al., in a clinical DSCT study in which stent lumen assessment of different sizes was investigated, showed that the negative predictive value was 100% for stents ≥3 mm and 90% for stents ≤2.75 mm [25]. Although stent diameters ≤2.75 mm lead to frequently false positive results, the authors drew the conclusion that DSCT coronary angiography could reliably rule out ISR irrespective of the stent size due to its high negative predictive value. In another clinical DSCT study all stent lumen with a diameter >3 mm were assessable, whereas only 81% of stents with a caliber of 3 mm could be evaluated [26].

To date, there is only one clinical pilot study assessing the diagnostic capability of the 256-MSCT for ISR detection in 28 patients [18]. In this study, Oda et al. attained a sensitivity and specificity of 100% and 55% by combining the CD kernel with an iterative reconstruction algorithm leading to positive and negative predictive values of 40% and 100%. Yet, the authors state the limitation that the diagnostic performance of the 256-MSCT in different stent sizes and types was not evaluated. In our in-vitro study, all stents with a diameter of 3 mm, that were reconstructed with the XCD kernel, showed an ALN that did not exceed 50%, which is sufficient to exclude a significant luminal stenosis >50%. Compared to group A stents (<3 mm), the extent of the halo effect was clearly less pronounced in group B (3 mm). Therefore, clinical in-vivo stent assessment might be possible for group B stents using the XCD kernel. However, future in-vitro and in-vivo studies are required to examine the possibility of stent lumen visualization for stents with a diameter of 3 mm in 256-MSCT.

Group C stents (>3 mm) revealed significantly lower ALN values and showed a negligible extent of the halo effect. These findings are in line with previously published literature and leads us to recommend 256-MSCT for the stent lumen assessment of coronary stents with a lumen diameter larger than 3.0 mm [1,11,21,26,27].

### Study limitations

Although the phantom was designed to mimic in-vivo conditions and has been used in a previous study, several limitations have to be considered.

First, all stents were scanned in an orientation parallel to the scanner’s z-axis. Previous studies have indicated that the quality of stent visualization may depend on the stent and scanner angulations [28,29].

Second, since a static phantom with an artificial continuous heart rate of 60 bpm was used, cardiac and respiratory movements could not be simulated. Therefore, high temporal resolution techniques of the 256-MSCT like fast rotation time and wide z-axis coverage were not regarded.

Third, the scan parameters in this study were chosen similar to the protocols for clinical routine. We did not evaluate the potential of advanced dose-saving algorithms for stent visualization. This may be an objective of further studies.

Fourth, the window settings were chosen in accordance with previous studies [1,30]. Yet, these settings indeed influence the visual measurement of the stent lumen diameter, as the visible strut thickness may vary at different settings.

Last, since the experimental set-up differed from other phantom studies concerning coronary stent visualization, the results are not fully comparable.

## Conclusions

With the 256-MSCT, the assessment of stent lumina larger than 3 mm is clinically possible if stent-dedicated kernels are applied. The XCD reconstruction kernel proved to be superior for lumen visualization independently from the stent caliber. The stent lumen visualization of stents with a diameter of 3 mm seems to be feasible but has to be validated in further studies.

Additional investigations are needed to assess the visualization of stents with a lumen diameter smaller than 3 mm. Further improvements in stent designs and materials as well as in CT spatial resolution and reconstruction methods could help accomplish the assessment of coronary stents using CT as part of the clinical routine.

### Availability of supporting data

The data sets supporting the results of this article are included within the article and its additional files.

## Abbreviations

ALN: Artificial lumen narrowing; BMS: Bare metal stents; CA: Coronary angiography; CAD: Coronary artery disease; CoCr: Cobalt chrome; CT: Computed tomography; DES: Drug eluting stents; DSCT: Dual source computed tomography; IQR: Interquartile range; ISR: In-stent restenosis; MSCT: Multislice computed tomography; PCI: Percutaneous coronary intervention; ROI: Region of interest; SiC: Silicon carbide; SLA: Stent lumen attenuation; TLA: Tube lumen attenuation.

## Competing interests

The authors declare that they have no competing interests.

## Authors’ contributions

FA and HS designed the trial, analyzed and interpreted the data and wrote the manuscript. FA performed the data acquisitions. DM contributed to the design of the study and drafted the manuscript. GM, WM and HUK have been involved in drafting and revising the manuscript. HK revised the manuscript critically and gave the final approval. HS also gave the final approval for this manuscript to be published. All authors read and approved the final manuscript.
